# Evaluation of machine learning models for personalized prediction of benefit from temporary mechanical circulatory support after out-of-hospital cardiac arrest

**DOI:** 10.1093/ehjdh/ztaf082

**Published:** 2025-07-18

**Authors:** Julian Kreutz, Jonathan Bamberger, Lukas Harbaum, Klevis Mihali, Georgios Chatzis, Nikolaos Patsalis, Mohamed Ben Amar, Styliani Syntila, Martin C Hirsch, Fabian Lechner, Bernhard Schieffer, Birgit Markus

**Affiliations:** Department of Medicine, Clinic for Cardiology, Angiology and Intensive Care Medicine, Philipps-Universität Marburg, Baldingerstraße, 35043 Marburg, Germany; Department of Medicine, Institute for Artificial Intelligence in Medicine, Philipps-Universität Marburg, Baldingerstraße, 35043 Marburg, Germany; Department of Medicine, Clinic for Cardiology, Angiology and Intensive Care Medicine, Philipps-Universität Marburg, Baldingerstraße, 35043 Marburg, Germany; Department of Medicine, Clinic for Cardiology, Angiology and Intensive Care Medicine, Philipps-Universität Marburg, Baldingerstraße, 35043 Marburg, Germany; Department of Medicine, Clinic for Cardiology, Angiology and Intensive Care Medicine, Philipps-Universität Marburg, Baldingerstraße, 35043 Marburg, Germany; Department of Medicine, Clinic for Cardiology, Angiology and Intensive Care Medicine, Philipps-Universität Marburg, Baldingerstraße, 35043 Marburg, Germany; Department of Medicine, Clinic for Cardiology, Angiology and Intensive Care Medicine, Philipps-Universität Marburg, Baldingerstraße, 35043 Marburg, Germany; Department of Medicine, Clinic for Cardiology, Angiology and Intensive Care Medicine, Philipps-Universität Marburg, Baldingerstraße, 35043 Marburg, Germany; Department of Medicine, Institute for Artificial Intelligence in Medicine, Philipps-Universität Marburg, Baldingerstraße, 35043 Marburg, Germany; Department of Medicine, Institute for Artificial Intelligence in Medicine, Philipps-Universität Marburg, Baldingerstraße, 35043 Marburg, Germany; Department of Medicine, Clinic for Cardiology, Angiology and Intensive Care Medicine, Philipps-Universität Marburg, Baldingerstraße, 35043 Marburg, Germany; Department of Medicine, Clinic for Cardiology, Angiology and Intensive Care Medicine, Philipps-Universität Marburg, Baldingerstraße, 35043 Marburg, Germany

**Keywords:** Out-of-hospital cardiac arrest (OHCA), Post-cardiac arrest syndrome (PCAS), Mechanical circulatory support (MCS), Machine learning, Mortality prediction

## Abstract

**Aims:**

The role of temporary mechanical circulatory support (tMCS) after out-of-hospital cardiac arrest (OHCA) remains controversial. This study evaluates machine learning (ML) models for predicting mortality and neurological outcomes, highlighting their potential as a tool to guide early tMCS decision-making.

**Methods and results:**

This retrospective study analysed five years of data from 564 adult non-traumatic OHCA patients treated at Marburg University Hospital. Four ML models (ANN, SVM, RF, XGBoost) were trained to predict in-hospital mortality and neurological outcome based on demographic, clinical, and treatment-related variables. Feature selection and SHAP analysis were used to optimize performance and identify patients potentially benefiting from tMCS. Overall, 144 patients (31.2%) out of 461 patients who fulfilled the inclusion criteria received tMCS: 39 left-ventricular microaxial flow pump, 76 venoarterial extracorporeal membrane oxygenation (VA-ECMO), and 29 biventricular support (ECMELLA). In 69 patients (14.9%) VA-ECMO implantation was performed as part of extracorporeal cardiopulmonary resuscitation. The survival rate of the tMCS group was 34.7% (50/144) compared to 52.7% (167/317) in the non-tMCS group. The highest predictive power for survival probability (with/without tMCS) could be achieved by XGBoost and RF when applied to the non-tMCS group. Machine learning identified 2.5% of non-tMCS patients likely to survive if treated with tMCS. In 23 (RF model) and 31 (XGBoost model) patients, the probability of survival increased by at least 5% with tMCS compared to their predicted outcome without tMCS. RF slightly outperformed XGBoost [area under the receiver operating characteristic curve (AUC) 0.85 vs. AUC 0.82].

**Conclusion:**

XGBoost and RF models accurately predict mortality and tMCS benefit in OHCA patients, supporting ML-based personalized therapy.

## Graphical Abstract

**Graphical Abstract:**
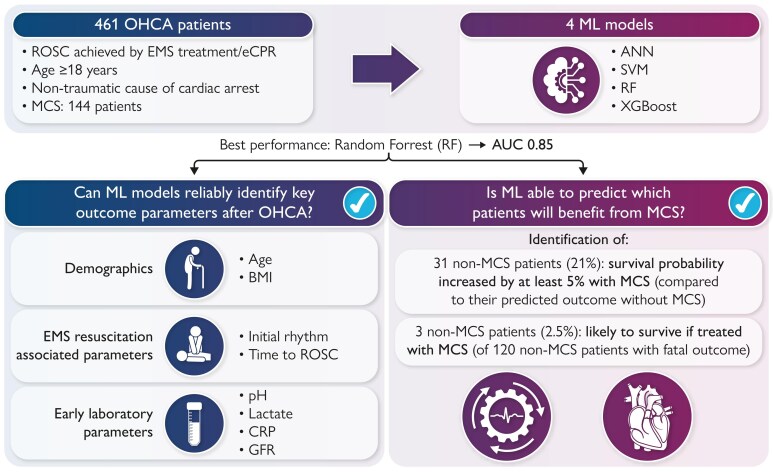

## Introduction

Out-of-hospital cardiac arrest (OHCA) is a leading cause of mortality worldwide, with survival rates remaining critically low despite advancements in emergency care and post-resuscitation strategies. Limited prognosis may be multifactorial and largely driven by the pathophysiology of post-cardiac arrest syndrome (PCAS), characterized by systemic inflammation, ischaemia-reperfusion injury, and persistent cerebral and myocardial dysfunction.^1,2^ These pathophysiological complexities pose significant challenges to therapeutic decision-making. This is particularly relevant with regard to the use of temporary mechanical circulatory support (tMCS), such as left-ventricular microaxial flow pump (mAFP), and venoarterial extracorporeal membrane oxygenation (VA-ECMO), which have gained increasing importance in managing refractory shock and haemodynamic instability in recent years.^3^ While extracorporeal cardiopulmonary resuscitation (eCPR) is one specific form of tMCS use during ongoing resuscitation, it can also be used during therapy to stabilize patients in post-resuscitation cardiogenic shock (CS). However, clearly defined, individualized criteria to guide the decision for or against tMCS and its selection and timing in the management of OHCA patients are currently lacking. Existing risk scores such as rCAST lack the precision required for individualized treatment decisions, particularly in the context of tMCS.^4,5^ Recent advances in artificial intelligence (AI) and machine learning (ML) have made them powerful tools for predictive analytics. The expanded options provided by such ML-based assessment tools make them particularly relevant in situations where rapid, time-critical decisions must be made.^6^ These models are capable of integrating various variables to identify treatment strategies and predict outcomes such as survival and neurological recovery after OHCA.^7–11^ Promising initial studies have demonstrated the potential of ML in identifying patients who may benefit from tMCS in the context of eCPR, with Li *et al*. showing its ability to identify high-risk patients and optimize intervention timing, particularly in underserved areas.^12–14^

The objective of this study is to evaluate whether ML-based models can provide incremental predictive value during tMCS in patients after OHCA, thereby informing and optimizing treatment strategies. By predicting mortality and neurological outcomes in OHCA patients, we seek to identify those most likely to benefit from tMCS, paving the way for a more personalized approach to care. The study provides an initial framework demonstrating that ML-driven approaches can outperform traditional scoring systems in guiding treatment decisions, particularly in critically ill OHCA patients requiring tMCS.

## Methods

### Study population and data collection

This retrospective study analysed data from non-traumatic OHCA patients admitted to the Cardiac Arrest Center (CAC) of the University Hospital of Marburg between January 2018 and December 2022. Inclusion criteria were either spontaneous return of spontaneous circulation (ROSC) or ROSC achieved by eCPR, age ≥ 18 years, and non-traumatic cause of cardiac arrest. Upon admission, all study patients required pharmacological circulatory support. Therefore, due to its lack of discriminative value, vasopressors and inotropes were not included as a separate variable for further analysis. Emergency medical services adhered to standardized protocols and used uniform equipment. The analysed data included demographics, pre-existing conditions, details of the cardiac arrest, initial treatments, and the use of tMCS. The analysis encompassed the entire cohort and differentiated between ‘survivors’ (discharged alive) and ‘non-survivors’ (those who died during hospitalization), as well as between patients who received tMCS (VA-ECMO and/or left-ventricular mAFP) during their hospital stay and those who did not (non-tMCS). To reflect real-world decisions, the models were based on variables available within the first hour of admission—when tMCS initiation is most relevant and rapid support is needed.

### Outcome measures

Primary endpoints were in-hospital mortality and neurological status at discharge, assessed using the Cerebral Performance Category (CPC) scale, ranging from 1 (good cerebral performance) to 5 (brain death). For evaluation, all patients were completely free from any form of sedative medication. Secondary endpoints included identifying patients who could potentially benefit from tMCS, as ML models predicted. In addition, ML models and logistic regression models were created to predict mortality and neurological outcomes, and the rCAST score was calculated and applied to each patient to compare the prognostic power of the predictors in the ML models.

### Statistical analysis

IBM SPSS Statistics (v29) and Stata (v16) were used for statistical analysis. Continuous variables were summarized as mean ± SD or median and interquartile range (IQR). Independent samples *t*-tests with Satterthwaite correction for unequal variances compared continuous variables. Chi-squared tests with Yates correction were used to analyse categorical variables and the Mann–Whitney *U* test was used for non-parametric data, directly comparing medians. The significance level was set at 0.05 for all tests. To explore predictors of outcome, a bivariate analysis was first performed comparing clinical and laboratory variables listed in *Table 1* between survivors and non-survivors. Variables with statistically significant differences were then entered into logistic regression models to identify independent predictors of survival and favourable neurological outcomes. The discriminatory ability of each model was assessed using the area under the receiver operating characteristic curve (AUC).

**Table 1:** Demographics, comorbidities, pre-hospital resuscitation-associated, and baseline laboratory parameters of the entire cohort and in ‘tMCS’ vs. ‘non-tMCS’ patients

	*n*=	All patients	tMCS	Non-tMCS	*P*-value
**Demographics and comorbidities**					
Number of patients^a^		461	144 (31.2)	317 (68.8)	
Age (years)^b^	461	65.7 (±14.3)	58.7 (±13.5)	68.8 (±13.5)	<0.001
Male sex^a^	461	334 (72.5)	113 (78.5)	221 (69.7)	0.051
BMI (kg/m^2^)^b^	306	28.5 (±5.7)	29.2 (±6.7)	27.9 (±4.9)	0.051
MI in the past/CHD^a^	430	81 (18.8)	17 (13.7)	64 (20.9)	0.083
Vitium of aortic/mitral valve (grade 2/3)^a^	430	32 (7.4)	3 (2.4)	29 (9.5)	0.013
Heart failure ≥ NYHA 3^a^	430	27 (6.3)	5 (4.0)	22 (7.2)	0.276
Atrial fibrillation^a^	430	54 (12.6)	8 (6.5)	46 (15.0)	0.016
Pacemaker^a^	430	14 (3.3)	3 (2.4)	11 (3.6)	0.766
Arterial hypertension^a^	430	219 (50.9)	54 (43.5)	165 (53.9)	0.051
Diabetes mellitus^a^	430	77 (17.9)	12 (9.7)	65 (21.2)	0.005
Nicotine abuse (> 5py)^a^	430	103 (24.0)	28 (22.6)	75 (24.5)	0.671
Alcohol abuse^a^	430	26 (6.0)	7 (5.6)	19 (6.2)	0.824
Hyperlipidaemia^a^	430	69 (16.0)	13 (10.5)	56 (18.3)	0.045
Chronic renal failure (KDIGO ≥ stage 3)^a^	430	43 (10.0)	2 (1.6)	41 (13.4)	<0.001
Renal replacement therapy^a^	461	16 (3.5)	2 (1.4)	14 (4.4)	0.167
COPD ≥ GOLD 2^a^	430	40 (9.3)	4 (3.2)	36 (11.8)	0.005
OSAS^a^	430	18 (4.2)	4 (3.2)	14 (4.6)	0.369
Bronchial asthma^a^	430	11 (2.6)	5 (4.0)	6 (2.0)	0.309
Pre-existing tracheostoma^a^	461	7 (1.6)	0 (0.0)	7 (2.2)	0.104
Cerebral stroke^a^	430	36 (8.4)	5 (4.0)	31 (10.1)	0.053
Thrombosis/PAE^a^	430	10 (2.3)	3 (2.4)	7 (2.3)	1.000
Malignant disease^a^	430	39 (9.1)	3 (2.4)	36 (11.8)	0.001
Peripheral arterial disease^a^	430	19 (4.4)	5 (4.0)	14 (4.6)	1.000
Carotid artery stenosis^a^	430	18 (4.2)	4 (3.2)	14 (4.6)	0.607
Hypo- or hyperthyroidism	430	29 (6.7)	8 (6.5)	21 (6.9)	0.534
**Resuscitation-associated parameters**					
Initial rhythm: shockable (VF/VT)^a^	461	193 (41.9)	72 (50.0)	121 (38.2)	0.017
Initial rhythm: asystole^a^	461	139 (30.1)	36 (25.0)	103 (32.5)	0.104
Initial rhythm: PEA^a^	461	129 (30.0)	36 (25.0)	93 (29.3)	0.336
Witnessed cardiac arrest^a^	461	319 (69.2)	97 (67.4)	222 (70.0)	0.565
Performed bystander CPR^a^	461	272 (59.0)	87 (60.4)	185 (58.4)	0.677
Resuscitation time (min)^b^	461	33.0 (±33.9)	62.7 (±44.4)	19.4 (±14.1)	<0.001
Mechanical CPR (chest compression device)^a^	460	93 (20.2)	79 (54.9)	14 (4.4%)	<0.001
**Baseline laboratory parameters (≤1 h after admission)**					
pH^c^	436	7.21 (7.04–7.31)	7.08 (6.86–7.26)	7.25 (7.12–7.32)	<0.001
Lactate (mmol/L)^c^	436	6.50 (3.60–11.88)	11.70 (6.68–16.00)	5.30 (2.90–9.30)	<0.001
CRP (mg/L)^c^	443	5.60 (2.00–20.50)	3.34 (1.40–10.08)	7.15 (2.63–30.23)	<0.001
GFR (mL/min)^c^	428	51.00 (37.00–66.00)	51.50 (41.25–62.00)	51.00 (35.00–68.00)	0.024

Baseline laboratory parameters were taken within the first hour after hospital admission.

BMI, body mass index; CRP, C-reactive protein; CHD, coronary heart disease; COPD, chronic obstructive pulmonary disease; GFR, glomerular filtration rate; MI, myocardial infarction; OSAS, obstructive sleep apnoea syndrome; PAE, pulmonary artery embolism; py, pack years; PEA, pulseless electrical activity; tMCS, temporary mechanical circulatory support; VF, ventricular fibrillation; VT, ventricular tachycardia.

^a^ *n* (%).

^b^Mean (SD).

^c^Median (IQR).

### Machine learning models

All ML analyses were performed in Python 3.11. Machine learning models were implemented using the XGBoost and Scikit-learn libraries (random forests, cross-validation, performance metrics). Neural networks were created using TensorFlow. Other packages used for data handling and visualization included SHAP, NumPy, Pandas, and Matplotlib. A dataset comprising 36 clinical and treatment-related variables (*Table 1*) for each patient was used to train four ML models: Artificial Neural Networks (ANN), Support Vector Machines (SVM), Random Forest (RF) and eXtreme Gradient Boosting (XGBoost).^15^ Feature selection followed an iterative elimination strategy for all models except ANN, aiming to balance model simplicity with predictive accuracy. A fivefold cross-validation approach ensured robust evaluation, minimized overfitting, and enhanced generalizability. Feature importance was further assessed using SHapley Additive exPlanations (SHAP) analysis to identify key predictors of mortality and neurological outcomes.^16^ Area under the receiver operating characteristic curve values and key predictors from the ML models were compared with those from logistic regression to assess consistency and potential added value in predicting survival and neurological outcomes. To assess model performance by type of circulatory support, additional subgroup analyses were performed for patients receiving mAFP, VA-ECMO, or ECMELLA. To explore potential differences in predictive performance, AUC values were calculated for subgroups defined by the form of circulatory support and timing of tMCS initiation (eCPR vs. post-ROSC). The core model was also incrementally expanded by adding features with moderate individual influence. Model performance was assessed using accuracy, precision, recall, F1 score, and AUC, with the latter serving as the primary metric for discriminating survivors from non-survivors. Model calibration was assessed using calibration curves, Brier score, and Expected Calibration Error (ECE). To further optimize model performance, an iterative backward feature elimination strategy was applied. Models were retrained with incrementally reduced or expanded feature sets, and their performance was systematically compared to identify the most robust feature combinations.

In addition, to estimate the potential benefit of tMCS in patients who did not receive circulatory support, the models were used to generate two outcome predictions for each non-tMCS patient, one assuming treatment with tMCS and one without. This individualized simulation approach enabled the identification of patients whose predicted likelihood of survival could have been improved with tMCS, supporting its potential utility as a decision-support tool in clinical practice. Finally, individual patient simulations were analysed to identify non-tMCS patients whose predicted outcomes changed from non-survival to survival when treatment with tMCS was assumed. Missing data were handled using the K-Nearest Neighbours (KNN) imputation technique, which ensured robust model performance by maintaining the integrity of the dataset. Several imputation methods were tested, with KNN providing the best results in terms of sensitivity, specificity, and accuracy. Little’s Missing Completely at Random (MCAR) test produced high *P*-values, indicating that the missing data were consistent with MCAR.

### SHAP interpretation

SHAP analysis was used to interpret feature importance, providing insight into how individual predictors influenced model predictions for both mortality and neurological outcomes. Thereby, the SHAP plots can be used to determine the contribution of each variable to the predictive power. The features are ranked in order of importance, with the top feature having the greatest impact on the model’s predictions. The models were further optimized by comparing the AUC for patients with and without tMCS, allowing for the identification of key predictors that influenced survival outcomes in tMCS patients. SHAP cohort bar plots were used to visually assess the differential impact of predictors on tMCS and non-tMCS patients.

### Ethical approval

The retrospective analysis was approved by the local Ethics Committee of the Philipps University of Marburg in accordance with the Declaration of Helsinki (ek_mr_14072021).

## Results

### Description of the overall cohort and comparison between temporary mechanical circulatory support and non-temporary mechanical circulatory support patients

**Figure 1:**
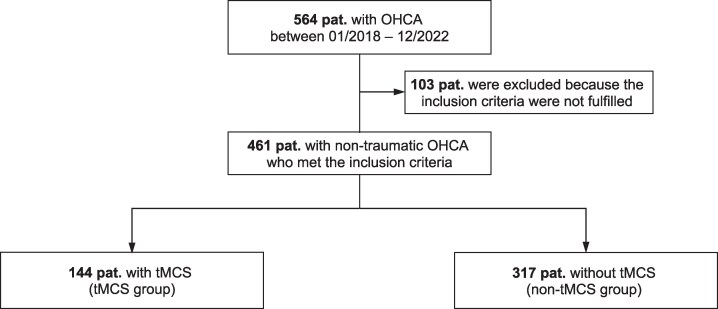
Study cohort. OHCA, out-of-hospital cardiac arrest; tMCS, temporary mechanical circulatory support.

During the observation period, 564 patients with OHCA were referred to the CAC of Marburg University Hospital (*Figure 1*). After excluding 103 patients due to failure to meet inclusion criteria, 461 patients were included in the final analysis. In this study, tMCS was used in 31.2% (*n* = 144) of patients, including 39 patients treated with a left-ventricular mAFP, 76 patients treated with VA-ECMO, and 29 patients treated with combined mAFP and VA-ECMO support (ECMELLA). Sixty-nine patients received VA-ECMO (alone or combined with mAFP) to provide eCPR. The remaining 68.8% (*n* = 317) of patients did not receive tMCS during hospitalization (non-tMCS group).

Overall, 34.7% (*n* = 50) of the tMCS patients survived to discharge, either home or to rehabilitation therapy, compared to 52.7% (*n* = 167) in the non-tMCS group. Survival rates varied significantly by type of tMCS: 64.1% (*n* = 25/39) survived with mAFP, 22.4% (*n* = 17/76) with VA-ECMO, and 27.6% (*n* = 8/29) with ECMELLA. Among the survivors, 70.0% (*n* = 152) demonstrated a favourable neurological outcome, with a CPC score of 1 or 2. Notably, 62.0% of tMCS patients (*n* = 31/50) achieved this outcome, compared to 72.5% of non-tMCS patients (*n* = 121/167), with no statistically significant difference observed (*P* = 0.157). Comparative analysis revealed significant differences in demographics and comorbidities between the tMCS and non-tMCS groups. Patients receiving tMCS were significantly younger (58.7 ± 13.5 vs. 68.8 ± 13.5 years, *P* < 0.001). They had a lower prevalence of comorbidities, including aortic/mitral valve disease grade 2/3 (2.4% vs. 9.5%, *P* = 0.013), atrial fibrillation (6.5% vs. 15.0%, *P* = 0.016), diabetes mellitus (9.7% vs. 21.2%, *P* = 0.005), chronic renal failure (KDIGO stage ≥ 3) (1.6% vs. 13.4%, *P* < 0.001), chronic obstructive pulmonary disease (COPD; ≥GOLD 2) (3.2% vs. 11.8%, *P* = 0.005), and malignancy (2.4% vs. 11.8%, *P* = 0.001).

In terms of pre-hospital resuscitation characteristics, patients in the tMCS group were more likely to present with an initial shockable rhythm (50.0% vs. 38.2%, *P* = 0.017). They had significantly longer resuscitation times to ROSC (62.7 ± 44.4 vs. 19.4 ± 14.1 min, *P* < 0.001). Laboratory tests performed within the first hour of hospital admission showed that tMCS patients had lower pH levels (7.08 [IQR 6.86–7.26] vs. 7.25 [IQR 7.12–7.32], *P* < 0.001) and higher lactate levels (11.70 [IQR 6.68–16.00] vs. 5.30 [IQR 2.90–9.30] mmol/L, *P* < 0.001) compared to the non-tMCS group. The results of demographics, comorbidities, resuscitation-related data, and baseline laboratory parameters are summarized in *Table 1*. Significant differences between the groups were also observed in diagnostic and therapeutic interventions. Whole-body CT imaging was performed in 85.9% of patients (84.0% in tMCS vs. 86.8% in non-tMCS, *P* = 0.436). ST-segment elevation myocardial infarction was observed more frequently in tMCS patients (29.2% vs. 12.3%, *P* < 0.001). Cardiac catheterization was performed in 93.8% of tMCS patients compared to 58.0% of non-tMCS patients (*P* < 0.001), with coronary interventions being more common in the tMCS group (61.8% vs. 31.5%, *P* < 0.001). Targeted temperature management (TTM, 34°C for 24 h) was applied more often in the tMCS group (82.6% vs. 54.6%, *P* < 0.001). Similarly, renal replacement therapy was required more frequently in tMCS patients (45.1% vs. 18.0%, *P* < 0.001). Among survivors, those in the tMCS group had longer in-hospital stays (median 19.0 days [IQR 17.0–27.0] vs. 15.0 days [IQR 11.5–19.5], *P* < 0.001) and longer duration of mechanical ventilation (median 364.0 h [IQR 241.0–508.0] vs. 187.0 h [IQR 139.5–301.0], *P* < 0.001). Maximum levels of neuron-specific enolase, indicative of neurological damage, were significantly higher in tMCS patients compared to non-tMCS patients (60.0 [IQR 42.0–92.0] μg/L vs. 29.0 [IQR 21.5–38.5] μg/L, *P* < 0.001), However, haemolysis-associated aspects triggered by tMCS should also be considered when interpreting these findings.

### Evaluation of machine learning models in predicting outcomes

**Figure 2:**
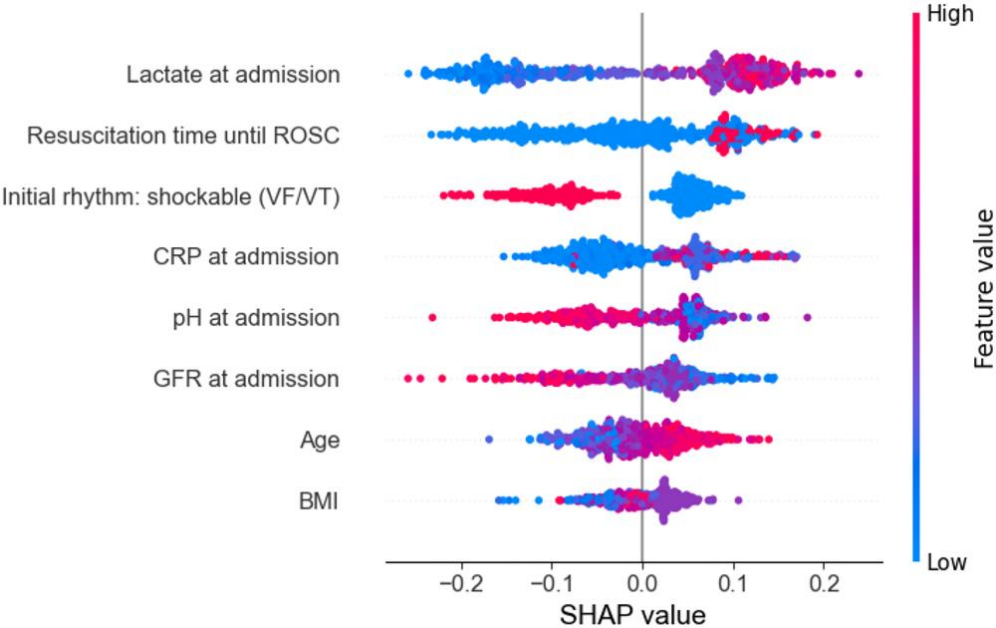
SHAP analysis of the best RF model for mortality prediction. Eight key predictors of mortality and neurological outcome in OHCA patients could be identified: lactate at admission, resuscitation time until ROSC, initial rhythm: shockable (VF/VT), CRP at admission, pH at admission, GFR at admission, age, and BMI. BMI, body mass index; CRP, C-reactive protein; GFR, glomerular filtration rate; VF, ventricular fibrillation; VT, ventricular tachycardia.

To evaluate whether ML models could outperform existing scoring systems, such as the rCAST score, and logistic regression models in predicting outcome after OHCA, different ML models were compared. Initial attempts using ANN and SVM yielded suboptimal results. Although the AUC values were higher than the rCAST score, the performance of ANN and SVM was still inferior when compared to other ML models, such as XGBoost and RF. Specifically, the RF model achieved an AUC of 0.88 using eight variables (XGBoost AUC 0.86; *Figure 2*) and an AUC of 0.85 using a reduced set of four blood parameters on admission [lactate, C-reactive protein, pH, glomerular filtration rate (GFR)] compared to an AUC of 0.80 for the rCAST score in predicting intrahospital mortality. In comparison, a logistic regression model including six variables [age, initial shockable rhythm (VF/VT), resuscitation time to ROSC, lactate, C-reactive protein, and GFR] achieved an AUC of 0.86 for overall survival and 0.84 for neurological outcome, highlighting the strong performance of both traditional and ML-based approaches. These findings closely align with previously published data from our group.^17^

For all ML models, the calibration curves showed good agreement between predicted and observed probabilities. To determine the contribution of each factor to its predictive power, the SHAP analysis of the best RF model identified eight important predictors that influence both mortality and neurological outcomes in OHCA patients (*Figure 2*). The SHAP analysis shows that ‘lactate on admission’ has the greatest impact on mortality after OHCA—higher and even average levels significantly increase the risk. Similarly, a longer ‘time to ROSC’ is associated with a worse prognosis, while lower levels of pH and GFR increase the risk of mortality. C-reactive protein has a clear dose–response relationship: the higher the value, the worse the prognosis; the absence of an initial shockable rhythm uniformly increases mortality. Age correlates directly with the risk of death, whereas average age has no significant effect. Body mass index (BMI) has a relatively small overall effect. These findings highlight the critical role of metabolic and clinical parameters in risk assessment after OHCA.

### Exploring the capacity of machine learning models to predict the impact of temporary mechanical circulatory support on patient outcomes

**Figure 3:**
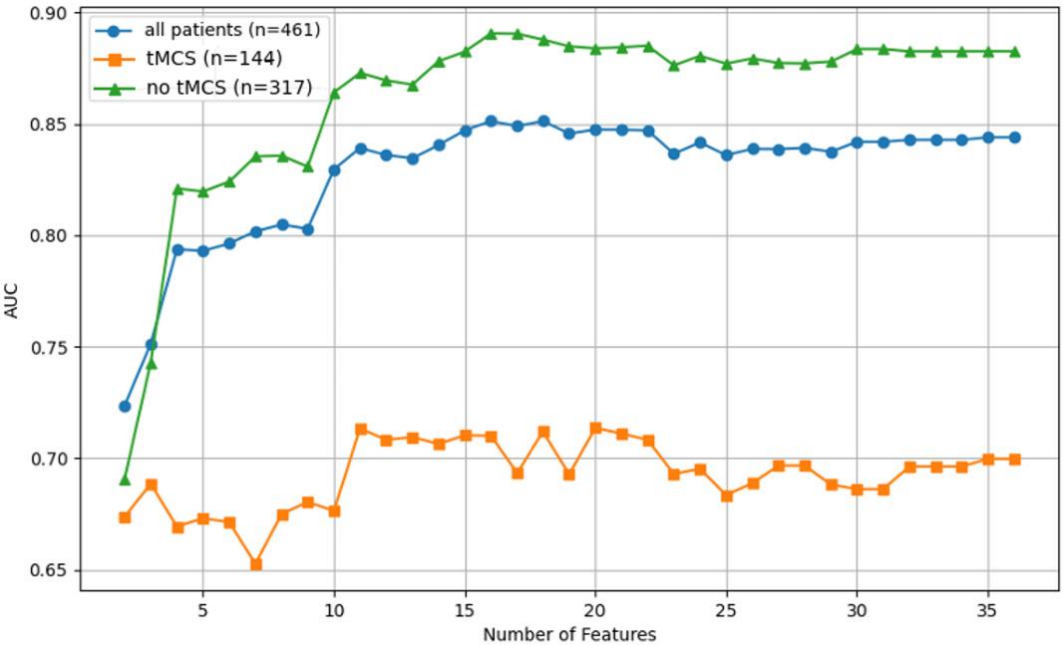
Illustrative example of AUC performance on the frequency imputation dataset across different tMCS groups using XGBoost. Overall cohort (circles), patients with tMCS (squares), and without tMCS (triangles). tMCS, temporary mechanical circulatory support.

To investigate whether ML could predict which patients might benefit from tMCS, we incorporated the variable ‘tMCS’ to generate dual predictions for patients who did not receive tMCS, allowing comparison of survival probabilities with and without tMCS. The best-performing models, XGBoost and RF, were used as the basis for further analysis. We applied an iterative feature removal strategy and optimized feature combinations to refine the models. In addition, SHAP cohort bar charts were used to compare feature influences in models with and without tMCS, providing further insight into the potential impact of tMCS on outcomes. Our analysis began by evaluating the XGBoost models, focusing on ensuring high AUCs for both tMCS and non-tMCS patients to minimize the difference between them while maximizing their combined values. Overall, the models produced satisfactory AUC values, but the AUC for the tMCS subgroup was significantly lower (*Figure 3*). This discrepancy suggests that the XGBoost models in their current form may be less suitable for accurately predicting the potential benefit of tMCS.

While models with more features demonstrated a more consistent AUC performance overall, tMCS patients consistently showed lower AUC values, even when a higher number of features were included. This difference may likely be due to the larger patient size of the non-tMCS group, which, to better optimize performance in this subgroup, preferably allows the boosting approach of XGBoost compared to the bagging approach used by RF. The SHAP analysis revealed that the impact of tMCS was significantly based on individual patient profiles and was less influenced by standardized predictors such as the initial shockable rhythm (VF/VT). Consequently, the final models were optimized using an extended set of parameters to better capture the complexity of the tMCS subgroup and to ensure robust performance across the entire cohort. The best XGBoost model achieved similar AUC values for the overall cohort compared to the best RF model. However, the AUC for patients with tMCS was comparatively lower, resulting in a notable performance difference between the tMCS and non-tMCS groups.

The simulation was performed for all patients to estimate their individual survival probability based on the respective model. In addition, for patients who did not receive tMCS, the models were used to simulate their survival probability assuming they had received tMCS. Of the 317 non-tMCS patients, many were predicted to have a mortality risk of <50%, meaning that they were expected to survive regardless of tMCS intervention. However, for 120 patients, the predicted risk of death without tMCS was above 50%, indicating that these patients were more likely to benefit from tMCS. Among these, the model identified three patients whose prognosis shifted from ‘death’ to ‘survival’ with tMCS, representing a potential survival improvement of 2.5% within the non-tMCS group.

Across all models tested, the RF model showed comparable performance to the XGBoost model, with a slight edge in predictive accuracy and a better balance of performance between tMCS groups. Specifically, the best XGBoost model with 13 features and KNN imputation achieved an AUC of 0.82 for the overall cohort (AUC tMCS = yes: 0.75; tMCS = no: 0.83) with an F1 score of 0.77 (the individual variables are listed in Supplementary material online, *Table S1*). In contrast, the RF model with 13 features achieved a higher overall AUC of 0.85 (*Table 2*), highlighting its superior predictive performance across all groups. The good calibration of the predicted survival probabilities was demonstrated by the low Brier scores and ECE values observed in the overall cohort. The 13-feature model had a Brier score of 0.136 and an ECE of 0.0395, the 11-feature model had a Brier score of 0.138 and an ECE of 0.0402, and the 8-feature model had a Brier score of 0.141 and an ECE of 0.0460. This transition to RF further highlighted its strengths, particularly in scenarios where a larger number of predictors are required to achieve optimal accuracy. The 8-feature RF model included the previously identified predictors except BMI, while the 11- and 13-feature models added additional variables (the individual variables are listed in Supplementary material online, *Table S2*). Although these extended models showed only marginally improved performance, the larger models overall gave slightly better results.

**Table 2:** Performance comparison of Random Forest models

RF model	AUC (all)	AUC (tMCS = yes)	AUC (tMCS = no)	Specificity	Sensitivity	F1 score
8 features	0.83	0.75	0.85	0.75	0.80	0.79
11 features	0.84	0.76	0.85	0.75	0.80	0.79
13 features	0.85	0.77	0.86	0.75	0.81	0.80

tMCS, temporary mechanical circulatory support.

To further explore potential differences within the tMCS cohort, additional analyses were performed for specific subgroups based on type of circulatory support. The best-performing RF models showed subgroup-specific AUCs of 0.72 (mAFP), 0.78 (VA-ECMO), and 0.81 (ECMELLA) for the 13-feature model, 0.69, 0.77, and 0.80 for the 11-feature model, and 0.70, 0.74, and 0.78 for the 8-feature model. SHAP analysis showed consistent feature importance across subgroups, except for pH, which was less relevant in mAFP patients. Model performance was also similar between patients who received MCS during CPR (eCPR) and after ROSC, suggesting that timing had little effect on predictive accuracy.

**Figure 4:**
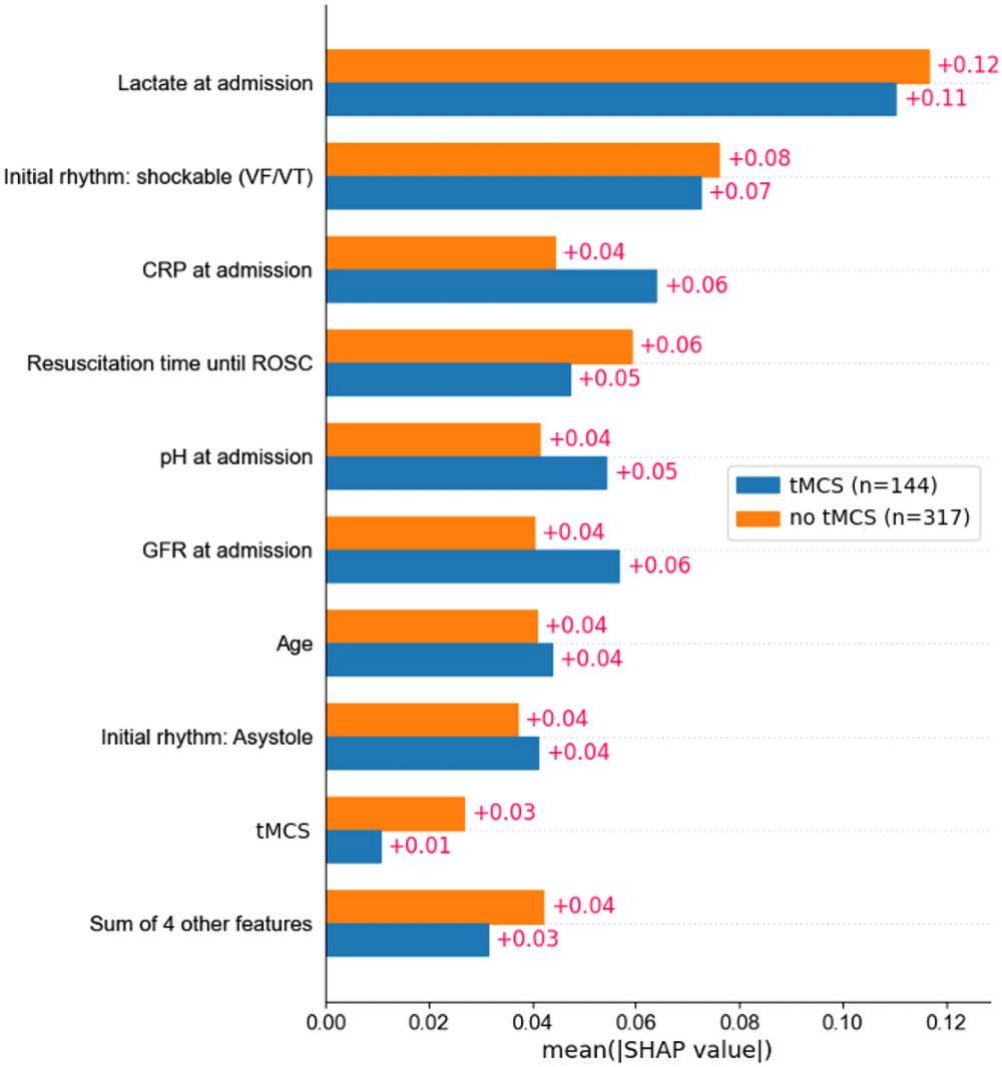
Comparison of feature influence on mortality prediction in tMCS and non-tMCS groups (SHAP, 13-feature RF model). The bar chart shows how the predictors affect patients with and without tMCS. The characteristics are also ranked by importance, and their influence on SHAP is directly visualized. For example, age has a consistent effect in both groups, while lactate and time to return of spontaneous circulation have a greater influence in the tMCS group than in the non-tMCS group; CRP and GFR on admission show a lesser influence on tMCS patients. The ‘Sum of 4 other features’ refers to the combined influence of the parameters: gender, mechanical CPR, arterial hypertension, and performed bystander CPR. CRP, C-reactive protein; GFR, glomerular filtration rate; ROSC, return of spontaneous circulation; tMCS, temporary mechanical circulatory support; VF, ventricular fibrillation; VT, ventricular tachycardia.

All models consistently identified two to three non-tMCS patients whose predicted outcome shifted from ‘death’ to ‘survival’ if treated with tMCS and ∼25 patients whose predicted probability of survival increased by at least 5% with tMCS. Of the five additional predictors [initial rhythm (asystole), gender, mechanical CPR, arterial hypertension, and performed bystander CPR], the parameter initial rhythm (asystole) has as much impact as the other four combined. These were added to the majority of the eight key predictors (lactate, time to ROSC, initial shockable rhythm, C-reactive protein, pH, GFR, and age) identified by SHAP analysis to refine the model’s performance. However, as shown in *Figure 4*, the combined effect of these predictors is still less than the individual effect of most of the eight key predictors. Nevertheless, these additional factors still contribute to a measurable overall improvement in performance.

## Discussion

To further improve outcomes after cardiac arrest, there is an urgent need for rapidly accessible and individually tailored diagnostics and therapeutic approaches. These can increasingly be provided by applying ML models during therapy. Owing to their ability to analyse large datasets and identify complex patterns, thus supporting real-time decision-making, they offer significant advantages over purely study-based methods. This study highlights the potential of integrating differentiated ML models in post-cardiac arrest management, particularly in enhancing the prediction of mortality and neurological outcomes compared to traditional scoring systems. Furthermore, according to our findings, ML may support the evaluation of the potential benefit of specialized therapy options like tMCS in CS and PCAS, which continues to be a significant challenge for intensive care unit teams today. This study was not designed to compare outcomes between different tMCS forms or with non-tMCS patients, as significant baseline differences, particularly in pH and lactate, would bias such analyses. The focus was on early ML-based prediction of individual benefit.

The application of AI and ML in clinical diagnostics and therapy has been increased in recent years. Various methods and techniques have been assessed in different contexts, including screening, therapy selection, and risk prediction.^18–22^ However, generally applicable ML-supported decision-making tools supporting OHCA management have not yet been established in daily clinical routine. While evaluating various established ML models (RF, XGBoost), this study identified demographic parameters (age, BMI), time-critical factors related to cardiac arrest (time until ROSC, initial shockable rhythm), and biomarkers of haemodynamics, inflammation, and organ function (lactate, pH, GFR, and C-reactive protein), which, when combined, offer superior predictive accuracy compared to traditional prognostic tools. Notably, as recently published by our group, these ML predictors were comparable to those identified through conventional statistical analysis, further validating used ML models.^17^ Additionally, a sub-analysis of the ECLS Shock trial, which included 78% of resuscitated patients, identified outcome-associated parameters using ML (XGBoost) that largely overlap with our predictors, further supporting the results of the present study.^23^ However, analysis of our patient cohort revealed that the decision against initiating tMCS was influenced not only by the study-defined predictors but also by patient-specific considerations, including advanced cognitive impairment and vascular comorbidities. Since Kresoja *et al*.^23^ demonstrated in their sub-analysis that neither tMCS nor tMCS-associated complications were the most influential predictors of mortality, it remains crucial to identify predictive factors that could guide a more beneficial use of tMCS. In this context, our results align with those of Li *et al*.,^14^ who investigated the role of AI in the selection, risk prediction, and monitoring of tMCS patient cohorts. However, while their study encompasses the entire continuum of care, our study specifically focused on identifying early predictors for patients most likely to benefit from tMCS. Indeed, the results of our study demonstrate that ML successfully identified non-tMCS patients who would have likely benefited from tMCS implantation, confirming its role in refining patient selection, and optimizing resource allocation in intensive care medicine. The recent ISHLT/HFSA guideline on acute tMCS recommends structured decision-making based on shock severity, pharmacological response, and end-organ perfusion.^24^ Our ML model, while not intended to replace such clinical judgment, can serve as a complementary tool by providing individualized survival predictions using early admission data. It is aligned with key guideline parameters—such as lactate, pH, and creatinine—and can help support timely decisions, particularly in complex or borderline cases.

Moreover, our ML-based approach differs from rule-based scores such as the ‘Durable MCS after ECLS calculator’ by Saeed *et al.*,^25^ which estimates prognosis after prolonged ECLS using predefined variables in a logistic regression model. In contrast, our model supports earlier decision-making using non-linear algorithms and only early available clinical data.

The deliberate focus on early-accessible parameters reflects the clinical urgency, as decisions regarding tMCS implantation often need to be made within critically narrow time frames. Although markers of multi-organ dysfunction are important for later prognostication, they typically emerge during later stages of intensive care and were therefore not included in this analysis. Similarly, detailed haemodynamic assessments and echocardiographic markers of ventricular function were excluded as these are often not available in the acute setting and would limit the applicability of the model for early decision-making.

Patients receiving tMCS were generally younger, more likely to be male, and more likely to have shockable rhythms and prolonged resuscitation, factors that are likely to have influenced clinical decision-making. Although accounted for in the ML models and reflected in the SHAP analysis, these real-world considerations underscore that ML should support—not replace—individual clinical judgment. Thus, these results further emphasize the importance of individualized precision medicine, particularly to ensure that highly specialized therapies like tMCS are used selectively to maximize benefits. These considerations are particularly relevant during ST-elevation myocardial infarction (STEMI), as this is often considered a reversible condition. Recent clinical trials such as DANGER Shock and previous findings of the working group around Kapur *et al*. highlight that early tMCS may benefit in selected STEMI patients in CS.^26–28^

One key consideration in the implementation of ML-based decision support is the selection of the most appropriate algorithm. In our study, RF showed the greatest stability in both tMCS and non-tMCS groups, likely due to its robustness to class imbalances, making it a more reliable choice for clinical applications. In contrast, while XGBoost effectively handles complex patterns, it is more prone to overfitting in unbalanced datasets, as seen in the tMCS vs. non-tMCS groups. The superior stability of RF in this context underscores its clinical utility in real-world applications. However, as the results of this study demonstrate, successfully integrating AI and ML into post-cardiac arrest management requires more than just developing robust algorithms. In addition to identifying established diagnostic markers, it also requires precisely determining the optimal timing of application and identifying more comprehensive parameters and innovative biomarkers—challenges that are likely even more complex than previously known. Consequently, the ML-based identification of predictors in the present study may represent, at most, an initial but valuable step towards precision medicine aimed at improving clinical outcomes.

For the effective implementation of ML methods in clinical practice, seamless integration into patient documentation systems and real-time monitoring systems seems essential. The incorporation of predictive analytics into decision support systems enables transparent, individualized risk assessments. By ensuring the consideration of ethical, regulatory, and data protection aspects, a safe and effective clinical application can be achieved.^29^

Ultimately, when integrated into clinical workflows, ML-based support can optimize the management of specialized therapies such as tMCS by simulating, for example, survival probabilities or haemodynamic changes with and without intervention. This facilitates more precise, data-driven treatment decisions and optimizes resource allocation. These findings highlight the need for further validation and seamless integration into clinical practice to fully leverage the potential of ML in intensive care medicine.

### Limitations

This study has several limitations that should be considered when interpreting the results. The single-centre, retrospective design may limit the generalizability of the findings to other healthcare settings or broader patient populations. The relatively small sample size, particularly within certain subgroups, may have affected the robustness of model training and validation, potentially leading to overfitting, or reduced predictive accuracy in external cohorts. Physician judgment, influenced by factors such as age, gender, initial rhythm, and resuscitation time, is likely to have influenced the use of tMCS and may have introduced selection bias not fully captured by the models. The dataset did not include systematic documentation of device-related complications (e.g. bleeding, limb ischaemia, and haemolysis), limiting our ability to assess potential harm or unnecessary use of tMCS on an individual level. In addition, imputation techniques were used to account for missing or incomplete data; however, this approach may introduce subtle biases that could affect model performance. The use of single-centre data may limit generalizability to broader clinical settings. Validation in larger, multicentre cohorts with more diverse populations is needed to confirm and refine the models. In addition, detailed information on shock phenotype, ventricular involvement (left, right, biventricular), and degree of cardiorespiratory compromise was not systematically included in the analysis. Future studies should address these aspects to allow more precise phenotype-based stratification and device selection. Finally, although the ML models demonstrated high predictive accuracy, their integration into routine clinical workflows remains a challenge.

## Conclusions

This study highlights the transformative potential of ML models to improve the management of OHCA patients, particularly in guiding the use of tMCS. By incorporating a comprehensive range of clinical variables, these models provided highly accurate and individualized predictions of survival and neurological outcomes, significantly outperforming traditional scoring systems. Their ability to simulate dual outcomes—with and without tMCS—offers a novel approach to support personalized treatment decisions, potentially optimizing resource allocation and patient outcomes. These findings highlight the need for further development and validation of these tools in larger, multicentre studies to confirm their clinical utility and ensure generalizability. Ultimately, the identification of individualized comprehensive parameters, the adequate timing of application, and the creation of intuitive, user-friendly platforms will be critical to integrating these predictive models into the clinical routine.

## Supplementary Material

ztaf082_Supplementary_Data

## Data Availability

The data underlying this article will be shared on reasonable request to the corresponding author.
