# Supplementary material for: Evaluation of machine learning models for personalized prediction of benefit from temporary mechanical circulatory support after out-of-hospital cardiac arrest
Source: Eur Heart J Digit Health. 2025 Jul 18;6(5):979–88. doi: 10.1093/ehjdh/ztaf082 (PMC12450520; doi:10.1093/ehjdh/ztaf082)
Supplement: ztaf082_Supplementary_Data [file ztaf082_supplementary_data.docx]

**Supplementary**

**Supplement Table 1.** Top- performing XGBoost model with 'MCS' variable (yes/no).

| **Feature Count** | **Features** |
| --- | --- |
|  |  |
| 13 | **Demographics and comorbidities:**  age, gender, arterial hypertension  **EMS resuscitation parameters:**  initial rhythm: shockable (VF/VT), initial rhythm: asystole, performed bystander CPR, resuscitation time until ROSC, mechanical CPR (use of chest compression device)  **Early laboratory parameters:**  pH at admission, lactate at admission, CRP at admission, GFR at admission  **MCS (yes/no)** |

**Supplement Table 2.** Top-performing RF models with 'MCS' variable (yes/no).

| **Feature Count** | **Features** |
| --- | --- |
| 13 | **Demographics and comorbidities:**  age, gender, arterial hypertension  **EMS resuscitation parameters:**  initial rhythm: shockable (VF/VT), initial rhythm: asystole, performed bystander CPR, resuscitation time until ROSC, mechanical CPR (use of chest compression device)  **Early laboratory parameters:**  pH at admission, lactate at admission, CRP at admission, GFR at admission  **MCS (yes/no)** |
|  |  |
| 11 | **Demographics and comorbidities:**  age  **EMS resuscitation parameters:**  initial rhythm: shockable (VF/VT), initial rhythm: asystole, performed bystander CPR, resuscitation time until ROSC, mechanical CPR (use of chest compression device)  **Early laboratory parameters:**  pH at admission, lactate at admission, CRP at admission, GFR at admission  **MCS (yes/no)** |
|  |  |
| 8 | **Demographics and comorbidities:**  age  **EMS resuscitation parameters:**  initial rhythm: shockable (VF/VT), resuscitation time until ROSC  **Early laboratory parameters:**  pH at admission, lactate at admission, CRP at admission, GFR at admission  **MCS (yes/no)** |
